# Orange corn diets associated with lower severity of footpad dermatitis in broilers

**DOI:** 10.1016/j.psj.2021.101054

**Published:** 2021-02-16

**Authors:** M.E. Abraham, S.L. Weimer, K. Scoles, J.I. Vargas, T.A. Johnson, C. Robison, L. Hoverman, E. Rocheford, T. Rocheford, D. Ortiz, D.M. Karcher

**Affiliations:** ∗ Department of Animal Sciences, Purdue University, West Lafayette, IN 47907-2050, USA; † Department of Animal and Avian Sciences, University of Maryland, College Park, MD, 20742, USA; ‡ Department of Animal Sciences, Michigan State University, East Lansing 48824-2604, USA; § NutraMaize, West Lafayette, IN 47906, USA; # Department of Agronomy, Purdue University, West Lafayette, IN 47907-2050, USA

**Keywords:** footpad dermatitis, orange corn, broiler, welfare, carotenoid

## Abstract

Footpad dermatitis (**FPD**), damage and inflammation of the plantar surface of the foot, is of concern for poultry because FPD affects the birds' welfare and production value. Footpad dermatitis is painful and causes costly chicken paw downgrades, carcass condemnations, and reduced live weights. However, a universal preventative has not been found. The hypothesis was that diets containing orange corn, when compared with diets containing yellow or white corn, would reduce the severity of footpad dermatitis in broiler chickens on wet litter. When compared with yellow and white corn, orange corn contains higher quantities of carotenoids, antioxidant pigments, believed to play a role in skin and feather health. This experiment was a randomized block, 3 × 2 factorial design: orange, yellow, and white corn diets with birds raised on wet or dry litter (control group). Female Ross 708 broilers (n = 960) were used to create 4 replicates of each diet x litter treatment combination. Footpads were scored at day 19, 27, 35, and 42, following the Global Animal Partnership standard's 0–2 scale of visual increasing severity: 0 indicates minimal damage and 1 and 2 indicate mild to severe lesions and ulceration, dark papillae, and/or bumble foot. At 42 d of age, birds on the wet litter had greater severity of FPD, scores 1 and 2, compared with the control group (88 vs. 13% respectively; *P* < 0.0001). At 42 d of age, prevalence of more severe footpad scores, 1 or 2, was lowest on the orange corn diet (33%), followed by white corn (56%) and yellow corn (63%). Birds fed the orange corn diet had higher BW throughout the study (*P* = 0.004) and had fat pads and livers with higher yellow pigment deposition (*P* < 0.005). Litter moisture content altered microbiome composition but corn type did not. In conclusion, the main determinant of FPD in this study was exposure to wet litter. When compared with yellow and white corn, orange corn was associated with improved bird growth and reduced severity of footpad dermatitis, especially at later time points.

## Introduction

Footpad dermatitis (**FPD**) is present in many different types of broiler production systems. In 1 study, Pagazaurtundua and Warriss (2006) estimated prevalence of FPD ranged from 9.6 to 98.1% depending on the housing system used. This common issue has a large impact on growers because the presence of FPD is associated with decreased live weight and leg meat yield and increased carcass condemnations (Hashimoto et al., 2013). These condemnations can have a massive economic impact. The chicken paw market alone comprises hundreds of millions of dollars of revenue. In total, southern China imports 300,000 tons of chicken paws each year, and in 2008, the Chinese chicken paw market was worth $280 million (Burgdorfer, 2009; Hornby, 2017). However, the impacts of FPD are not purely economic. Footpad dermatitis is a welfare concern for the birds experiencing the condition. Not surprisingly, the damage to the birds' feet causes alterations in gait and is associated with decreased BW gain and reduced feed intake resulting from pain (Haslam et al., 2007; Shepherd and Fairchild, 2010; Da Costa et al., 2014). The importance of footpad scoring is evidenced by its presence in many welfare audits in the United States and Europe.

The pervasiveness of this condition makes FPD an important area for research, and FPD has been studied extensively to determine its causes and possible preventative measures. Conventional approaches to the study of FPD have included looking at nutritional, environmental, and genetic correlations with FPD severity. Many possible predisposing factors of FPD, such as litter quality, litter depth, water drinker type, bird age, and bioavailability of biotin in the diet (Pagazaurtundua and Warriss, 2006), have been identified. Yet, Jong et al. (2014) found the largest contributing factor or cause to be the presence of wet litter. Preventatives for FPD must have practical routes of administration, so feed and water additives have commonly been tested. Previously tested dietary preventatives include vitamins (biotin, riboflavin, pantothenic acid), minerals, amino acids (methionine and cystine), feed-grade enzymes, electrolytes (Na, K), and microelements (Zn) (Shepherd and Fairchild, 2010; Swiatkiewicz et al., 2017).

Despite the many additives or products tried, a definitive solution to prevent FPD in broilers has yet to be identified. Carotenoids may be part of a solution as they have shown to have protective effects in birds. Carotenoids are a group of natural pigments responsible for the yellow, orange, and red coloration of fruit, flowers, and vegetables. Like humans, birds cannot synthesize carotenoids; they rely solely on dietary intake of carotenoids (Goodwin, 1992).

In humans, increased dietary intake of carotenoids results in elevated blood plasma/serum and tissue carotenoid levels. These increased levels of carotenoids have been associated with reduced incidence of several chronic diseases, including cardiovascular diseases (Li and Xu, 2015; Leermakers et al., 2016), type 2 diabetes (Hamer and Chida, 2007), several types of cancer (Ge et al., 2013; Zhou et al., 2016), and reduced overall mortality (Buijsse et al., 2005; Zhao et al., 2016). Carotenoid-related health benefits have mainly been attributed to their antioxidant, antiapoptotic, and anti-inflammatory properties (Krinsky and Yeum, 2003; Bohn, 2019). Many of these antioxidant and anti-inflammatory properties have recently been linked to carotenoids' effects on intracellular signaling cascades, which influence downstream gene expression and protein translation (Kaulmman and Bohn, 2014). Carotenoid supplementation reduced redness in the human skin exposed to UV light and conferred protection against certain skin tumors in rats (Mathews-Roth and Krinsky, 1985; Lee et al., 2000; Juturu et al., 2016; Grether-Beck, 2017). Dietary supplementation of carotenoids has also produced health benefits for poultry and therefore has potential utility within the poultry industry. Bédécarrats and Leeson (2006) reported that the carotenoid lutein improved and modulated the birds' immune systems; they saw increased antibody titers and an improved vaccine response when increased lutein levels were present in the diet.

In broilers challenged with coccidia, a diet high in carotenoids reduced the incidence of FPD and digital ulcers in the coccidial-challenged treatment group and the control group (Nogareda et al., 2016). These studies suggest that carotenoids aided the effectiveness of the immune system and also provided protective skin effects to counteract damage to the birds' feet.

Carotenoids have a multitude of downstream effects on the bird's microbiome and lifetime productivity. In broilers, carotenoid supplementation improved meat quality; in layers, carotenoid supplementation increased both egg production and individual egg weight (Marounek and Pebriansyah, 2018). A meta-analysis reported that dietary supplementation of the carotenoid canthaxanthin in laying hens improved feed intake, feed conversion ratio (**FCR**), egg production, egg weight, and egg yolk mass (Umar Faruk et al., 2018). Carotenoid supplementation can alter the intestinal microbiome, or microbial community that resides in the intestinal tract. In pigs, while the microbial community alpha diversity was unchanged, dietary β-carotene supplementation altered the membership of the microbiome of weaning (Li et al., 2019) and finishing pigs (González-Prendes, 2019). Carotenoid supplementation likely affects the microbiome of poultry as well, but little research has elucidated specific details of these changes (Gong et al., 2020). Further research is necessary to identify the effects of carotenoids on the microbiome.

Poultry producers commonly supplement carotenoids using feed additives in the diet. This results in more intensely colored egg yolks in layers or a more intense yellow color to the skin in broilers. Carotenoids in feed additives are derived from extracts of natural sources such as marigolds or peppers or from artificial carotenoids obtained from genetically bioengineered sources such as corn (Grashorn, 2016; Moreno et al., 2016; Díaz-Gómez et al., 2017). Traditional breeding and selection for natural variation has created specific corn genotypes that contain higher levels of carotenoids and are much more orange in color than conventional yellow dent corn (Egesel et al., 2003; Harjes et al., 2008; Heying et al., 2014; Ortiz et al., 2016). Orange corn contains total carotenoid levels of 40 to 60 μg/g, whereas conventional yellow corn averages ∼10 to 20 μg/g.

Orange corn was originally biofortified through natural breeding to deliver increased nutrition (increased provitamin A carotenoids) to humans in the developing world. Biofortification is a feasible and cost-effective process of increasing the density of vitamins and minerals in a crop through plant breeding (Bouis and Saltzman, 2017). Biofortified orange corn used for dietary supplementation of xanthophylls in poultry feed diets has been shown to increase yolk color (Ortiz et al., 2020). However, carotenoid deposition from consumption of non-genetically modified organism (**non-GMO)** orange corn and the potential protective effects for birds' feet has not been studied in broiler chickens. The present study sought to use a wet litter challenge, known to cause FPD, to evaluate orange corn's possible protective effects on broiler chicken feet, and to evaluate carotenoid deposition through altered pigmentation of the liver and fat pad. Cecal microbiome analysis was completed to evaluate differences between wet and dry litter as well as among 3 corn types.

## Materials and methods

### Study Design

Female Ross 708 chickens (N = 960, 40 birds per pen) were placed in 3.71 m^2^ pens on wood shavings at a stocking density of 0.08 m^2^/bird at 1 d of age and followed up through 42 d of age. The project was designed as a 3 × 2 factorial arrangement of a randomized complete block. The birds were raised on 1 of 2 litter treatments, dry or wet litter, and fed 1 of following 3 diets: 1) orange, 2) yellow, or 3) white corn. Each diet × litter treatment combination was replicated 4 times with 1 empty pen between each of the experimental pens. All pens began the study with fresh pine wood shavings litter. The wet litter was achieved by wetting the litter by hose once/week to reach at least 60% moisture content (determined by the litter's appearance and consistency; Welfare Quality score 2 [leaves imprint of foot and will form a ball if compacted, but does not hold its form; Welfare Quality, 2009]). Throughout the duration of the study, no decaking was necessary in either litter treatment group and the dry litter stayed dry for the duration of the 42-day study. Within each pen, feed and water were provided ad libitum via 1 bell drinker and 1 feeder, this equated to 5-cm feeder space and 5-cm water space per bird. Supplemental pan feeders were provided during the first 5 d of the study. Feed was formulated to Ross 708 specifications, differing only in the type of corn, and was fed in 3 phases: 0 to 13, 14 to 27, and 28 to 42 d of age (Tables 1 and 2). Mortality was recorded daily. The project and procedures were reviewed and approved by the Purdue University Animal Care and Use Committee (Protocol#:1803001706).

**Table 1:** Ingredient composition and formulated analysis for 3 phase diets fed to female broilers.

Ingredient (%)	Phase 1 (0–13 d)	Phase 2 (14–27 d)	Phase 3 (28–42 d)
Corn^1^ (orange, yellow, white)	57.66	63.76	66.90
Soybean meal (48%)	35.27	29.68	26.30
Soybean oil	3.00	3.00	3.52
Salt	0.48	0.46	0.48
DL-methionine	0.24	0.21	0.12
L-lysine HCl	0.11	0.10	0.02
L-threonine	0.06	0.04	–
Calcium carbonate	1.41	1.38	1.49
Monocalcium phosphate	1.42	1.02	0.82
Vitamin–mineral premix^2^	0.35	0.35	0.35
Formulated composition			
ME (kcal/kg)	3,095	3,161	3,220
CP	21.47	19.21	17.68
Methionine	0.56	0.50	0.39
Lysine	1.25	1.09	0.93
Calcium	0.96	0.85	0.84
Phosphorus	0.68	0.58	0.53

1 Diets were formulated to match Ross 708 specifications. Nutrient content of each of the different corn types (orange, yellow, white) was assumed to be equivalent for diet formulation.

2 Vitamin and mineral premix supplied the following per kilogram of diet: vitamin A, 5484 IU; vitamin D3, 2643 ICU; vitamin E,11 IU; menadione sodium bisulfite, 4.38 mg; riboflavin, 5.49 mg; d-pantothenic acid, 11 mg; niacin, 44.1 mg; choline chloride, 771 mg; vitamin B12, 13.2 μg; biotin, 55.2 μg; thiamine mononitrate, 2.2 mg; folic acid, 990 μg; pyridoxine hydrochloride, 3.3 mg; I, 1.11 mg; Mn, 66.06 mg; Cu, 4.44 mg; Fe, 44.1 mg; Zn, 44.1 mg; Se, 300 μg.

**Table 2:** Nutritional analysis of different corn sources fed to female broilers during a 42-day grow out period.

Analysis (%)	Analyzed corn			Formulated corn specifications
	Orange	Yellow	White	
ME (kcal/kg)^1^	3,792	3,770	3,748	3,373
CP	10.70	7.68	8.92	7.5
Crude fat	5.50	3.62	3.72	3.5
Methionine	0.31	0.53	0.56	0.18
Lysine	0.23	0.23	0.26	0.24
Phosphorus	0.33	0.36	0.31	0.28
Calcium	0.01	0.01	0.03	0.01

1 ME acquired through direct calculation.

### Corn Analysis

#### Nutritional Analysis

All corn sources were submitted to Midwest Laboratories Inc. (Omaha, NE) for analysis. ME was measured through direct computerized calculation by Midwest Laboratories Inc. using the formula ME= (96-(0.202∗P))/(100∗DE) where P is protein and DE is digestible energy. CP and crude fat were calculated by Association of Official Analytical Chemists methods 990.03 and 2003.05, respectively. Methionine and lysine were calculated by Association of Official Analytical Chemists methods 994.12 Alt. I and III, respectively. Phosphorus and calcium were calculated through the Association of Official Analytical Chemists 985.01 (mod) method.

#### Carotenoid Analysis

All sample preparations and extractions were performed under yellow light to minimize photoisomerization reaction. Moisture content on corn was determined following the American Association of Cereal Chemists recommendations (AACC, 1983). Carotenoid extractions were performed following a procedure described by Ortiz et al. (2020). Carotenoid analysis was completed with a Shimadzu HPLC Prominence UFLC XR series coupled with a diode array detector. Carotenoids separations were achieved using a YMC C30 (3 μm 2.0 mm × 150 mm column) with a YMC carotenoid guard column (2.0 mm × 23 mm). Detailed chromatographic conditions used were previously reported by Ortiz and Ferruzzi (2019). Carotenoids were identified by comparing spectral information in the literature (Britton et al., 2004) and retention times with authentic all-trans-carotenoid standards. Quantification was based on 7-point calibration curves prepared spectrophotometrically from authentic all-trans standards with a concentration range between 0.01 and 7.67 μmol.

### Bird Weight and Footpad Scores

Pen weights were taken at diet changes; average BW, FCR, and weight gain were calculated. Average BW was calculated by taking the weight of the pen and dividing by the number of birds in that pen. Feed conversion ratio was calculated by dividing feed consumed per pen by weight gained for each pen during a given period. Feed weights were recorded before placement in feeders in each pen. Footpad dermatitis scoring followed the Global Animal Partnership guidelines (GAP guidelines, 2017). This scoring system is a 0–2 scale of visually increasing severity where scores of 1 and 2 represent mild to severe lesions and ulceration, dark papillae, and/or bumble foot, which is considered FPD. A score of 0 was considered ideal and represented minimal to no damage to the foot. Based on Welfare Quality guidelines (Welfare Quality, 2009), footpad scores were taken on approximately 30% of each diet x litter treatment combination, which equated to 8 random birds per replicate (N = 192). However, owing to labor limitations during the day 42 sampling, 2 birds per replicate were sampled instead of 8 (N = 48).

### Microbiome Library Preparation and Analysis

At 42 d of age, broilers were harvested, and cecal contents were collected from 4 birds per pen (96 birds total) and immediately stored at 4°C. Cecal metagenomic DNA was isolated using the Qiagen (Valencia, CA) MagAttract PowerMicrobiome DNA/RNA kit following the manufacturer's instructions. A Qiagen TissueLyzer was used for the bead beating step. Extracted DNA was used for the construction of a 16S rRNA gene library following a standardized protocol (Kozich et al., 2013). Briefly, Illumina indexed reads were created using PCR amplification of the V4 region of bacterial 16S rRNA gene. Amplification was determined through gel electrophoresis. Using water as the DNA template, no PCR amplicons were observed in the negative control. Amplified DNA was normalized using the SequalPrep Normalization Plate (Invitrogen) and pooled into a single library for each 96-well plate. Library concentration of four 96-well pools were determined using the KAPA Library Quantification Kit (Roche), and library average fragment length was determined using the Bioanalyzer (Agilent) with a high-sensitivity kit. After the confirmation of proper DNA concentration, the pooled samples, mock community, and water were sequenced (Illumina, MiSeq v2 kit, 500 cycles). Sequences were demultiplexed as per oligonucleotide bar code sequence with Illumina software. Sequences were deposited in the National Center for Biotechnology Information Sequence Read Archive database under Bioproject PRJNA669652.

The analysis of 16S amplicon sequences was carried out with QIIME2, version 2019.1. Briefly, low-quality sequences were removed with DADA2 (Callahan et al., 2016), with the first 13 bases of the forward and reverse reads being removed owing to low quality. Samples were rarefied and sampled at 5,035 sequences per sample.

### Colorimeter Readings

After cecal collections, fat pad and liver samples were taken from 32 birds per diet × litter treatment combination (n = 8 per replicate) at 42 d of age. CIELab color measurement was performed in fat pad and liver samples with a portable Konica Minolta CR-400 Chroma Meter (Konica Minolta Sensing Americas, Inc., Ramsey, NJ). The CIELab color space expresses color as 3 values: L∗ for the lightness from black (0) to white (100), a∗ from green (−) to red (+), and b∗ from blue (−) to yellow (+). Yellow pigment deposition was of interest for this study, so only the yellow/blue or b∗ score will be presented here. A higher b∗ score indicates more yellow coloration present in the organ of interest. a∗ scores were not statistically significant and L∗ scores were minimally significant for orange vs. white least squares means differences. As such a∗ and L∗ scores are not presented here.

### Statistical Analysis

#### Production and Bird Characteristics

The PROC MIXED procedure of SAS 9.3 (SAS/STAT User's Manual, 2014) with type 3 fixed effects was used to analyze average bird weight, consumption, FCR, and fat pad and liver colorimeter scores. Main and interaction effects of the diet, litter treatment, and age of bird were tested for bird weight, consumption, and fat pad and liver colorimeter scores. Main and interaction effects of diet and litter treatment were evaluated for FCR. Footpad scores were evaluated with Kruskal-Wallis tied rank scores. Least squared means were calculated for footpad score, average bird weight, and fat pad and liver colorimeter scores. Results are presented as least squares means with pooled SEM and *P*-value *P* < 0.05 designated as significant.

#### Microbiome

For microbiome analysis, the pen was considered the experimental unit, so pen replicates were merged into a combined pen sample with equal representation from all animals from the same pen. After this, a phylogenetic approach was used to characterize alpha and beta diversity. Alpha diversity metrics were analyzed for normality using Shapiro's test (shapiro.test function) in R v3.6.1, and all were found to be normally distributed. ANOVA testing (aov function) followed by Tukey's post hoc test (TukeyHSD function) were run with litter and diet as the main effects as well as their interaction. Amplicon sequence variants (**ASV**) were classified with the 99% clustered Silva database (version 132) (Quast et al., 2012) that was trained with the primers from this experiment. DESeq2 v1.26 (Love et al., 2014) (function DESeq in R) was used to determine differentially abundant genera as per the diet and litter treatment on a pairwise basis. QIIME2 output files were imported into R with the qiime2R package v0.99.20 to generate figures and to read in data files. For reproducibility, QIIME2 commands and R scripts are available at www.github.com/john2929/OrangeCorn.

## Results

### BW, Feed Consumption, FCR, and Diet Analysis

BW was higher in birds fed the orange corn diet throughout the study (diet × phase effect, *P* = 0.004; Table 3). Litter treatment did not have an effect on BW (*P* > 0.05). At the end of phase 1, the average weights of white diet–fed birds were 0.40 kg, while those fed yellow and orange diets were 0.41 kg. At the end of phase 2, yellow and white corn–fed birds weighed 1.23 kg, while orange corn–fed birds weighed 1.28 kg. On day 42, the end of phase 3, yellow corn–fed birds weighed 2.29 kg, white corn–fed birds weighed 2.35 kg, and orange corn–fed birds weighed 2.46 kg. No differences in overall FCR were observed for diet, litter, or diet × litter treatment interaction effects (Table 4; *P* > 0.05). There were diet effects on FCR during phase 1 and phase 3, and litter treatment effects for FCR during phase 3 only (*P* < 0.05). Only phase and litter × phase effects were observed for feed consumption (Table 3; *P* < 0.05).

**Table 3:** BW and feed consumption^1^ during a 42-day grow out with female broilers fed 3 different corn sources over 3 phases of diets.

Diet	Phase 1 0–13 d	Phase 2 14–27 d	Phase 3 28–42 d	Overall
	BW (kg)			
Orange corn	0.41^a^	1.28^a^	2.46^a^	
Yellow corn	0.41^a^	1.23^a^	2.29^c^	
White corn	0.40^a^	1.23^a^	2.35^b^	
Pooled SEM	0.02	0.02	0.02	
Wet litter	0.40	1.25	2.34	
Control litter	0.41	1.25	2.39	
Pooled SEM	0.02	0.02	0.02	
	Feed consumption^2^			
Orange corn	1.30	1.75	1.54	1.53
Yellow corn	1.28	1.63	1.67	1.53
White corn	1.33	1.69	1.66	1.56
Pooled SEM	0.06	0.06	0.06	0.03
Wet litter	1.29^a^	1.63^a^	1.70^a^	1.54
Control litter	1.31^a^	1.76^a^	1.54^b^	1.54
Pooled SEM	0.05	0.05	0.05	0.03

	ANOVA probability	
	BW	Feed consumption
Diet	0.0004	0.7331
Litter treatment	0.2574	0.9831
Phase	<0.0001	<0.0001
Diet x litter	0.4564	0.2574
Diet x phase	0.0044	0.2573
Litter x phase	0.3623	0.0103
Diet x litter x phase	0.7122	0.5791

1 Weights and feed consumption are written as LS means with pooled SEM below each column. ^a,b^Means in the same column within each classification bearing different letters are significantly different (*P* < 0.05).

2 Feed consumption represents average feed consumed in kilograms per bird.

**Table 4:** FCR^1^ during a 42-day grow out with female broilers fed 3 different corn sources over 3 phases of diets.

Diet	Phase 1 0–13 d	Phase 2 14–27 d	Phase 3 28–42 d	Overall
	FCR			
Orange corn	1.13^b^	2.10^a^	1.32^b^	1.56
Yellow corn	1.19^a,b^	1.99^a^	1.60^a^	1.67
White corn	1.21^a^	2.03^a^	1.49^a,b^	1.64
Pooled SEM	0.02	0.10	0.06	0.04
Wet litter	1.19^a^	1.96^a^	1.57^a^	1.65
Control litter	1.15^a^	2.12^a^	1.37^b^	1.60
Pooled SEM	0.02	0.08	0.05	0.03
	Phase ANOVA probability			
Diet	0.0428	0.7367	0.0093	0.0992
Litter treatment	0.1595	0.1556	0.0058	0.2032
Diet x Litter	0.1146	0.2301	0.5992	0.0782

Abbreviation: FCR, feed conversion ratio.

1 FCR is written as LS means with pooled SEM below each column. ^a,b^Means in the same column within each classification bearing different letters are significantly different (*P* < 0.05).

There were noticeable nutritional differences among corn sources. These differences were not evaluated statistically but represent practical differences. The orange corn had the highest levels of ME, CP, and crude fat (Table 2). Total carotenoid levels were 0.57, 5.36, and 21.03 μg/g for white, yellow, and orange corn, respectively (Table 5). White corn did not contain any carotenes and contained the lowest levels of carotenoids measured. Yellow corn contained an intermediate level of all measured carotenoids relative to white and orange corn. Orange corn had the highest levels of all carotenoids. Quantities of zeaxanthin, β-cryptoxanthin, total xanthophylls, carotenes, and provitamin A (**PVA**) carotenoids in orange corn were noticeably higher than the quantities found in yellow or white corn.

**Table 5:** Carotenoid composition (μg/g) of sourced corn used in diets fed to female broilers during a 42-day grow out.

Source of corn^2^	Carotenoid type (μg/g)^1^										
	LUT	ZEA	ACRYP	BCRYP	15BC	13BC	BC	XAN	CAR	PVAC	TOTAL
White	0.37	0.21		0.06				0.57	0.00	0.01	0.57
Yellow	3.18	1.74	0.22	0.28	0.13	0.15		5.27	0.09	0.16	5.36
Orange	3.41	13.49	0.51	1.95	0.50	0.51	0.67	19.35	1.68	2.15	21.03

1 Carotenoid types were measured using HPLC and are as follows: LUT, all-*trans*-lutein; ZEA, all-*trans-*zeaxanthin; ACRYP, alpha-cryptoxanthin; BCRYP, beta-cryptoxanthin; 15BC,15-*cis*-beta-carotene; 13BC,13-*cis*-beta-carotene; BC, all-*trans*-beta-carotene; XAN, total xanthophylls; CAR, total carotenes; PVAC, pro-vitamin A carotenoids; TOTAL, total carotenoids.

2 Corn values are an average of samples taken from sourced corn used at each diet mixing for the 3 phases.

### Litter Moisture Altered Microbiome Composition

**Figure 1:**
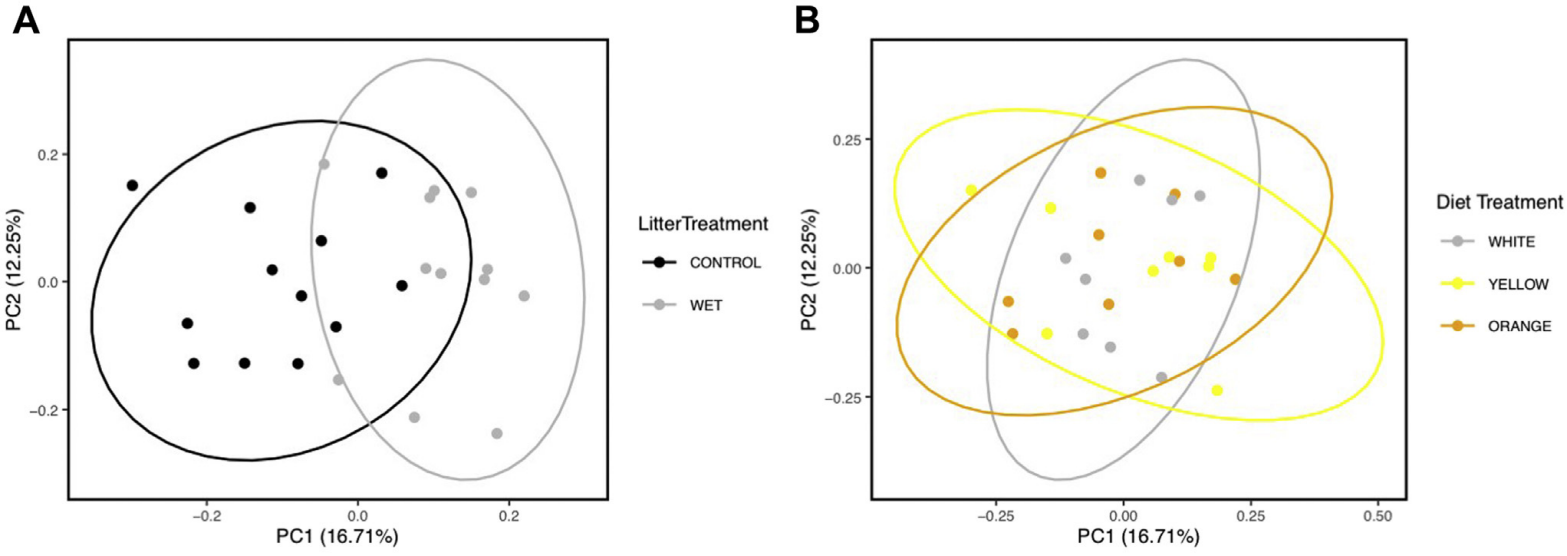
Principal coordinates analysis based on Bray-Curtis dissimilarity matrix. Samples are colored as per (A) the litter type the broilers were housed on or (B) the corn type in the diet. The cecal microbiome community was statistically different as per litter treatment when distances were calculated with Bray-Curtis, weighted UniFrac and unweighted UniFrac (PERMANOVA, q < 0.05). Ellipses indicate a 95% CI of the range of individual samples.

**Figure 2:**
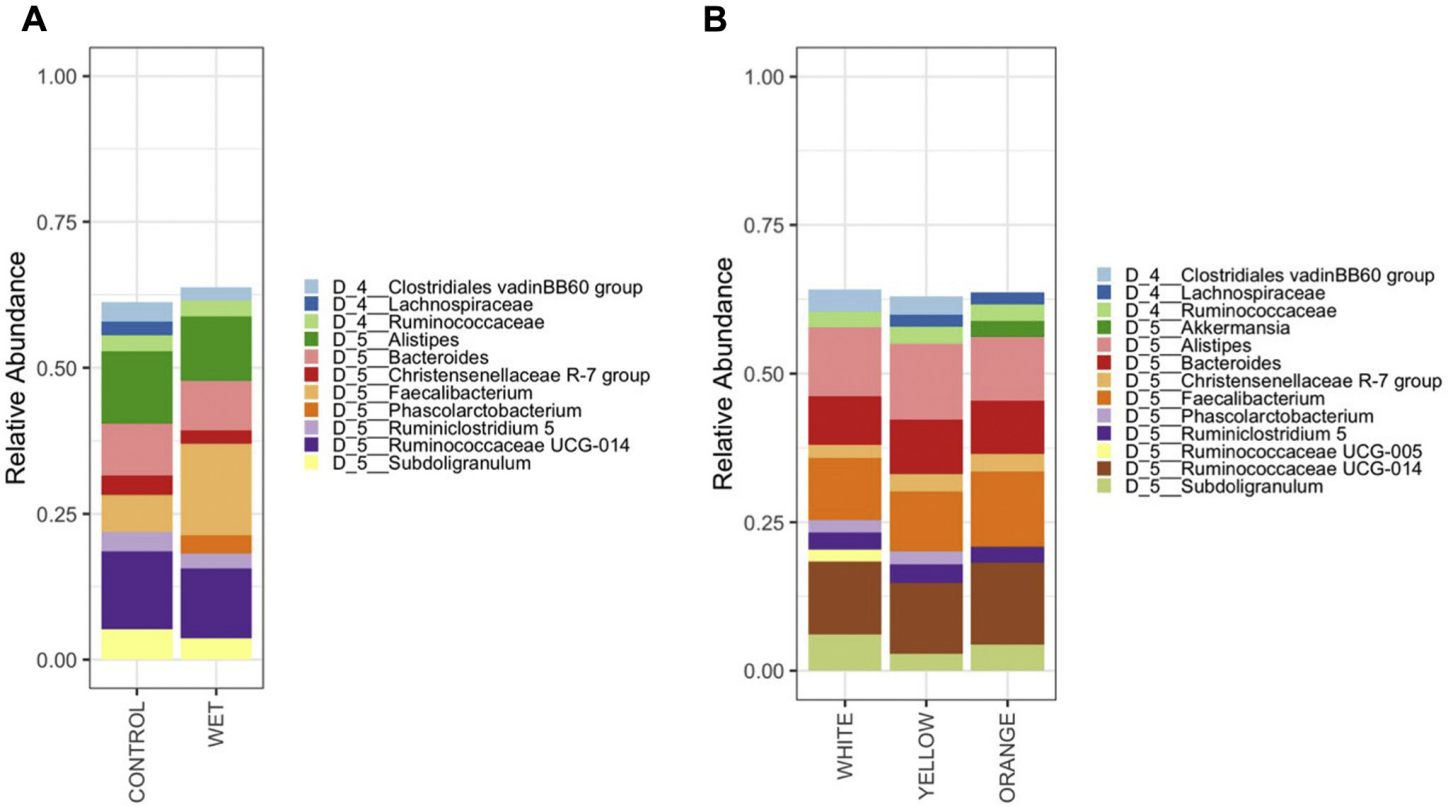
Average relative abundance according to (A) litter treatment or (B) diet of members of the microbiota classified at the genus level. A total of 24 pens were used in this experiment divided evenly between the diet x litter treatment groups. ASV genus taxonomic classifications are proceeded with “D_5.” Classifications beginning with “D_4” indicate ASV classified at the family level but unable to be classified further at the genus level. Abbreviation: ASV, amplicon sequence variant.

**Figure 3:**
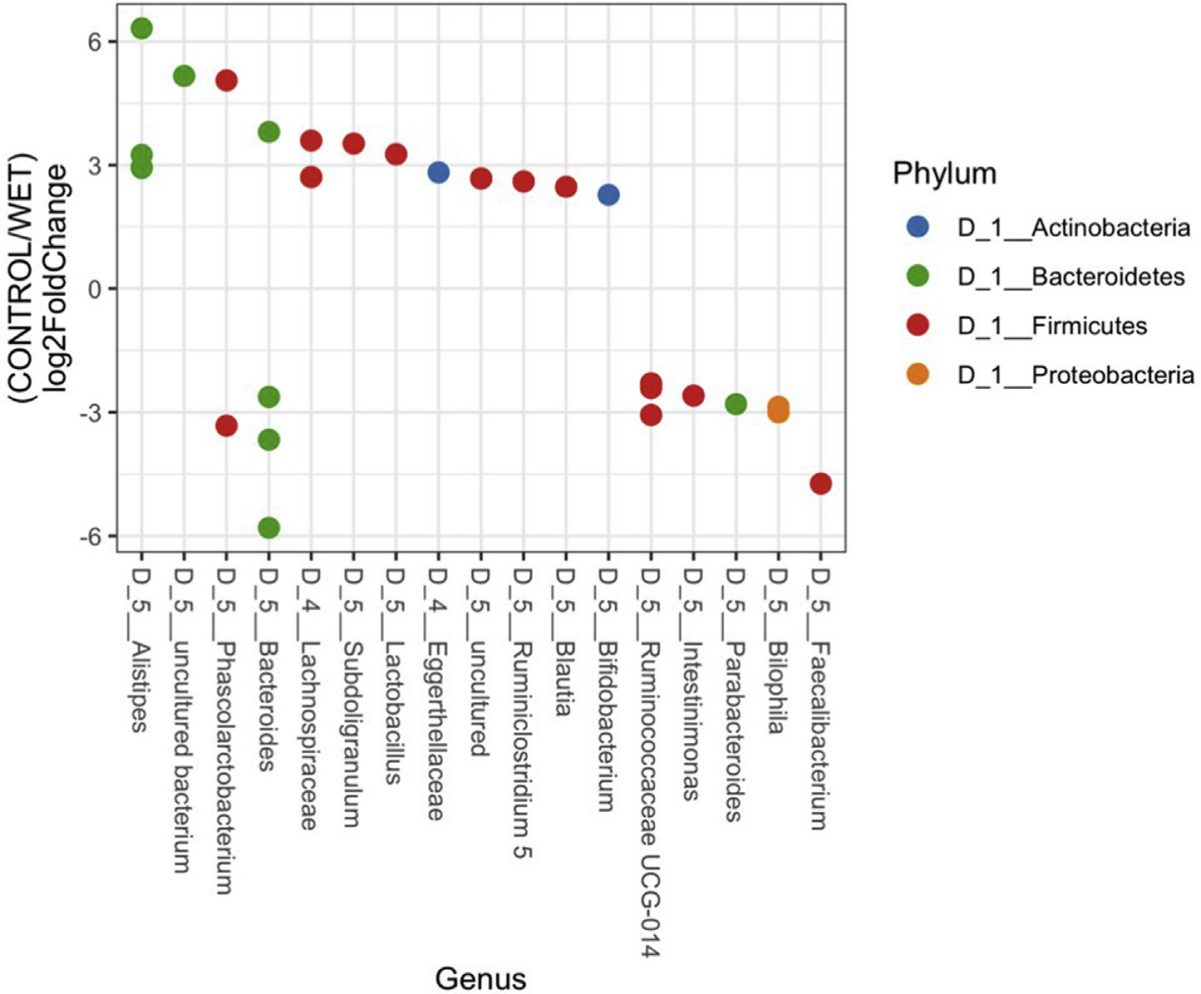
Log_2_ fold change in ASV compared between the control litter and wet litter groups. Log_2_ fold change values greater than 0 indicate the fold change increase in the control samples, while log_2_ fold change values less than zero indicate the fold change decrease in the control litter samples. Each dot represents a single ASV. Samples are colored according to their phylum assignment. ASV genus taxonomic classifications are proceeded with “D_5.” Classifications beginning with “D_4” indicate ASVs classified at the family level but unable to be classified further at the genus level. Abbreviation: ASV, amplicon sequence variant.

Litter moisture content was the major driver of community type (Figure 1A). Differences in community composition by Bray-Curtis (F-statistic = 1.5), weighted UniFrac (F-statistic = 2.2), and unweighted UniFrac (F-statistic = 1.4) all found that cecal microbial communities from chickens raised on wet and dry litters were different (PERMANOVA, q < 0.05). In addition, broiler chicken cecal microbial ASV evenness and Shannon diversity of ASV were decreased in birds raised on wet compared with dry litter (ANOVA, *P* < 0.05).Observed number of ASV and Faith phylogenetic diversity were not different between birds on wet and dry litter (ANOVA, *P* > 0.05). *Faecalibacterium* increased as a genus in relative abundance in the broilers raised on wet (16% of the community) compared with dry (7% of the community) litter (Figure 2). As per the differential abundance test, DESeq2, sequence variants from the genera *Alistipes* and *Faecalibacterium* were enriched profoundly on dry and wet litters, respectively (Figure 3). In addition, ASV from 15 other genera were also significantly altered owing to litter moisture (Figure 3).

### Orange Corn Altered Microbiome Composition

**Figure 4:**
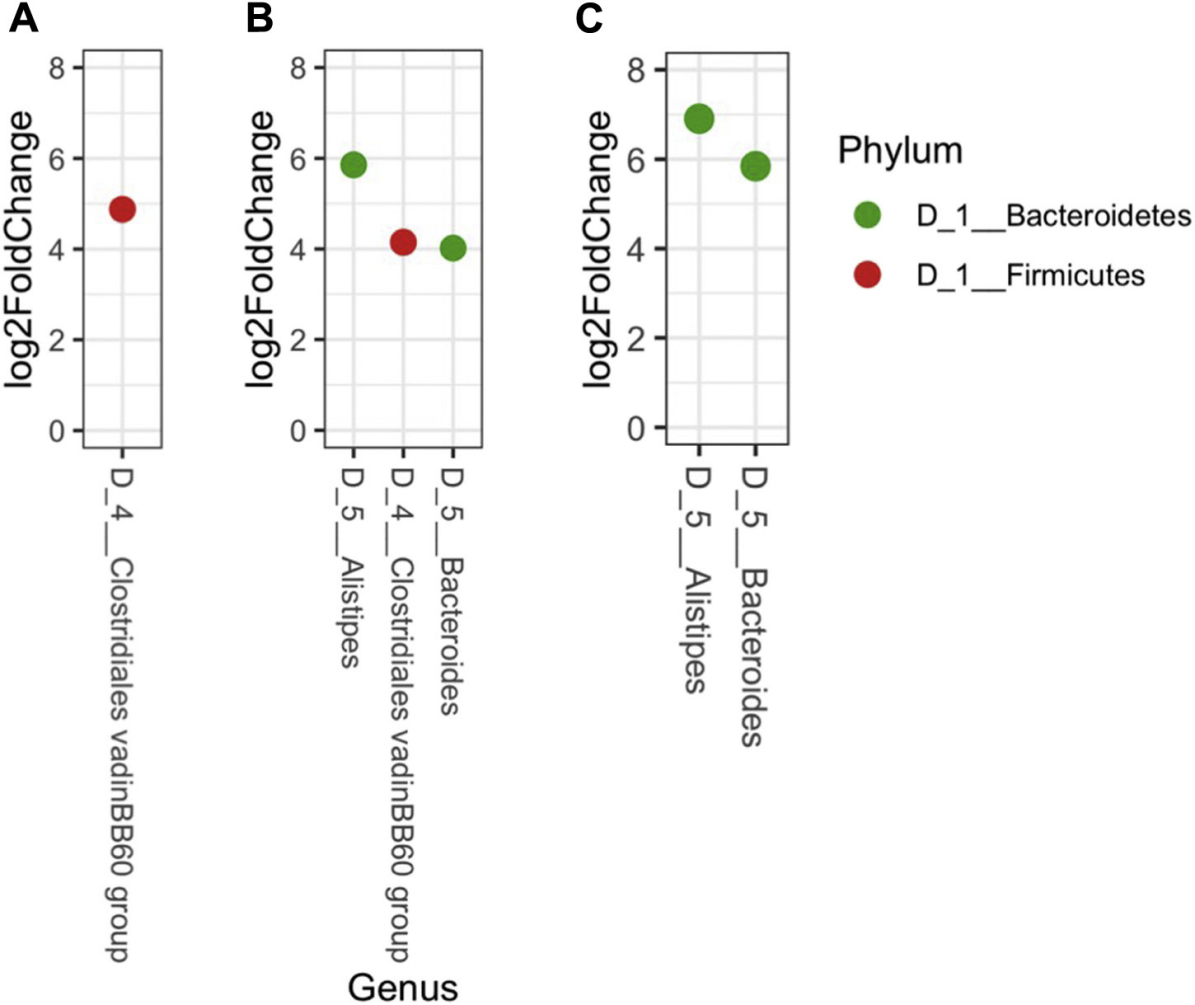
Pairwise log_2_ fold change in ASV compared between the diet groups. Log_2_ fold change values greater than zero indicate an increase in the (A) white compared with yellow, (B) white compared with orange and (C) yellow compared with orange corn groups. Each dot represents a single ASV. Samples are colored as per their phylum assignment. ASV genus taxonomic classifications are proceeded with “D_5.” Classifications beginning with “D_4” indicate ASV classified at the family level but unable to be classified further at the genus level. Abbreviation: ASV, amplicon sequence variant.

Diet type did not change the overall composition of the cecal microbiome as a main effect (Figure 1B; PERMANOVA, q > 0.1), and the interaction between diet and litter treatments was not significant (PERMANOVA, q > 0.1). Diet did not alter the alpha diversity of the chicken cecal microbial community (ANOVA, *P* > 0.1). The interaction between diet and litter treatments was significant for Faith's phylogenetic diversity (*P* = 0.36), but no pairwise post hoc comparisons were significant (data not shown). There were some individual taxa differences owing to diets. An ASV assigned to the *Clostridiales* vadinBB60 group was decreased in the yellow (30-fold decrease) and orange (16-fold decrease) corn diet groups compared with the white group (Figure 4). In addition, there was a decrease in 1 ASV assigned to each of *Bacteroides* (>8-fold) and *Alistipes* (>60-fold) in the orange corn–fed group compared with the white and yellow corn–fed groups (Figure 4).

### Footpad Scores

**Figure 5:**
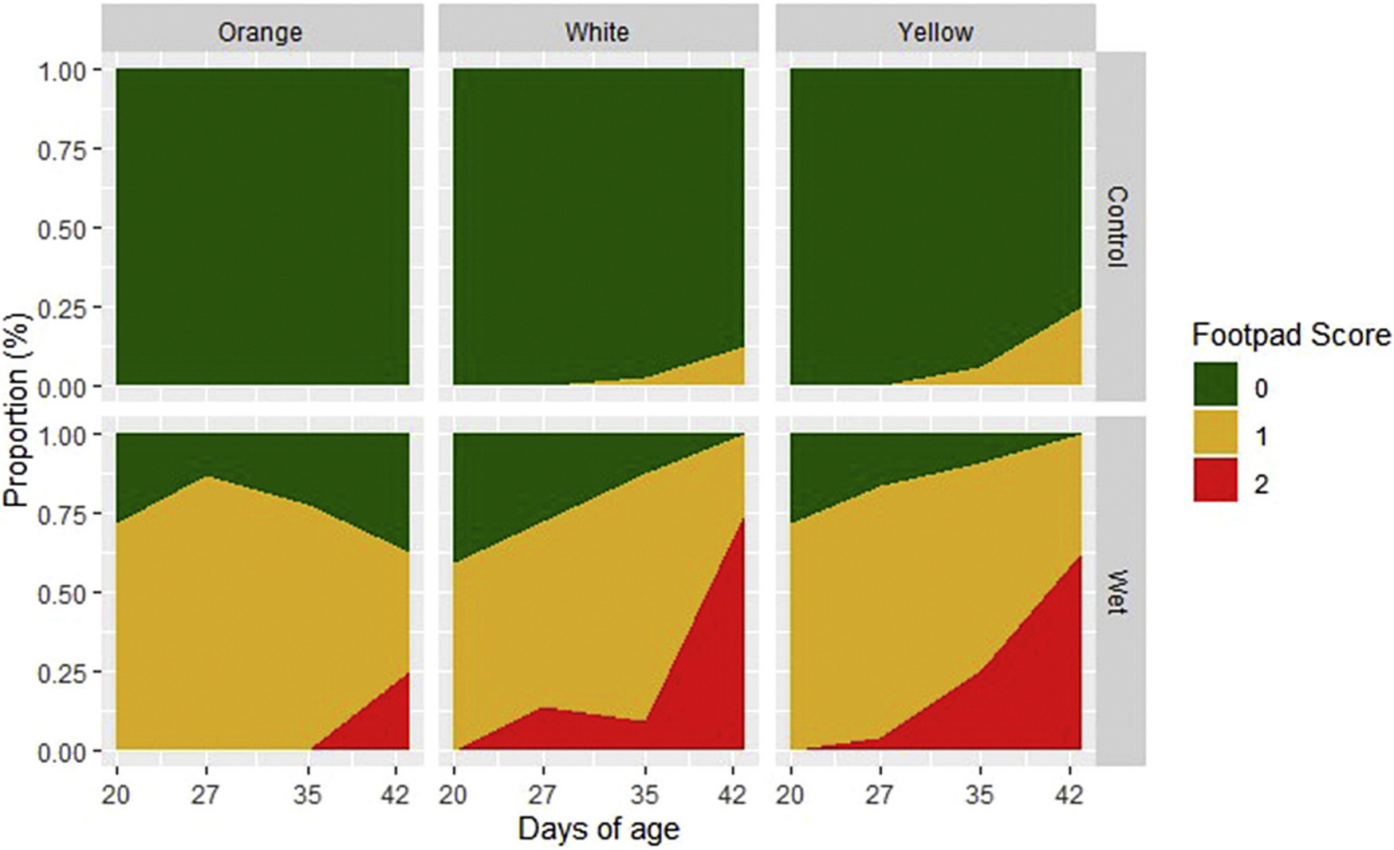
Proportions of broiler footpad scores throughout the 42-day grow period. Scores were taken at 20, 27, 35, and 42 d of age on approximately 30% of birds in each diet x litter treatment group (N = 192; except N = 48 for day 42). Scores of 1 and 2 indicate mild to severe lesions and ulceration, dark papillae, or bumble foot and the presence of footpad dermatitis. Scores of 0 are ideal and represent minimal changes to the foot. Green is best followed by yellow then red. D 27 and 35 represent differences (*P* < 0.05) for the wet litter treatment group.

The overall prevalence of moderate (score 1) to severe (score 2) FPD on wet litter and control litter was 78 and 2%, respectively (Figure 5). At 42 d of age, birds on wet litter had more FPD 1 and 2 scores than the control group (88 vs. 13%, respectively; *P* < 0.0001). Diet differences were only significant (*P* < 0.05) on day 27 and 35. Diet effects (Figure 5) were as follows: though not statistically significant, on day 20, birds fed orange and yellow corn diets had similar prevalence of moderate to severe FPD (36%, n = 64) which was slightly higher than the birds fed the white corn diet (30%, n = 64). At day 27 (*P* < 0.05), prevalence of moderate to severe FPD was lowest in the white corn group (37%, n = 57) followed by the orange (51%, n = 39) and yellow corn groups (63%, n = 32). On day 35 (*P* < 0.05), birds fed orange corn had the lowest moderate to severe FPD (38%, n = 63), followed by the birds fed the white (45%, n = 63) and yellow (48%, n = 64) corn. At 42 d of age, though not statistically significant, the prevalence of birds with footpad scores of 1 or 2 was least on the orange corn diet (33%, n = 15), followed by white corn (56%, n = 16) and yellow corn diets (63%, n = 16). Within the control litter group, only the birds of the orange corn group had an absence of FPD throughout the entire study. As the birds grew, both white and yellow corn groups had increasing incidence of moderate and severe footpad scores on wet litter. The orange corn group on wet litter saw a peak of moderate footpad scores at 27 d of age which then declined until termination of the study. The orange corn diet had the longest absence of severe footpad scores, the wet litter group fed yellow and white corn had severe footpad scores by 27 d of age, while birds fed the orange corn did not develop severe scores until 35 d of age. All 3 corn groups saw increasing severe footpad scores between day 35 and 42 with the largest increase occurring in the white corn group followed by the yellow and orange corn groups, respectively.

### Pigment Deposition

**Figure 6:**
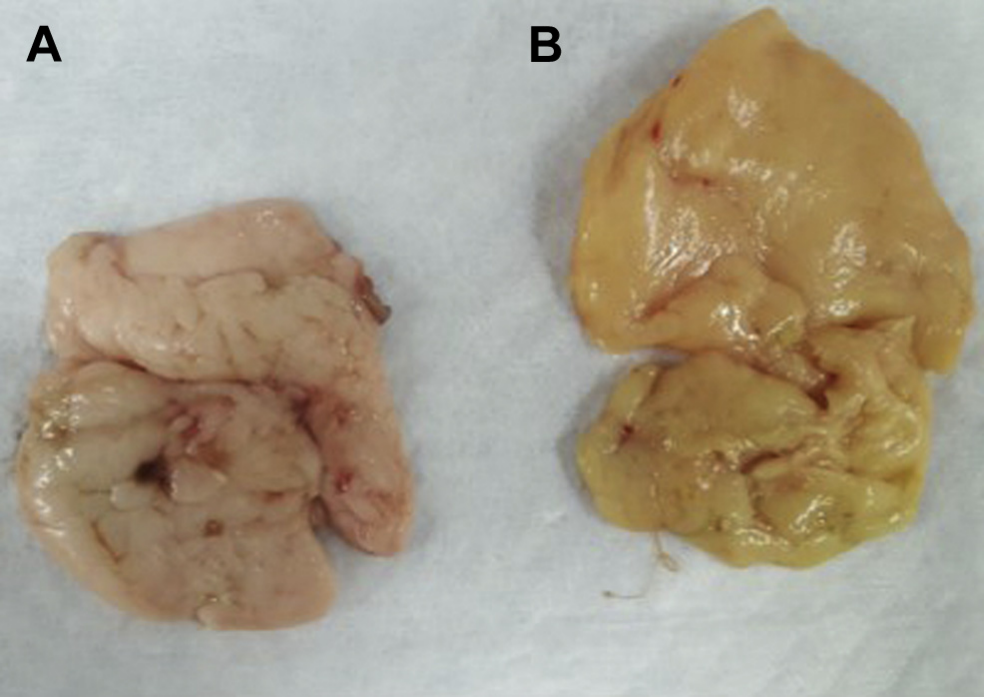
Fat pads of 42-day-old broilers fed white (A) or orange (B) corn throughout the duration of the study.

Yellow pigment deposition (b∗) in fat pads and livers was higher in birds fed the orange corn diet compared with those fed the white and yellow corn diets (*P* < 0.005, Figure 6), which were measured at harvest, 42 d of age (Table 6). Fat pad yellow pigment deposition (b∗, unitless) was highest in birds fed the orange corn diet (23.40) followed by yellow (17.81) and white (10.39) corn diets. Similarly, liver yellow pigment deposition was highest in birds fed the orange corn diet (7.13) followed by the yellow (5.18) and white (4.21) corn diets.

**Table 6:** Yellow pigment deposition in the liver and fat pad represented by the b∗ score of the colorimeter measured at the end of grow out, 42 d of age, in female broilers.

Diet	Mean b∗ color score^1^^,^^2^	
	Liver	Fat pad
Orange corn	7.13^a^	23.40^a^
Yellow corn	5.18^b^	17.81^b^
White corn	4.21^b^	10.39^c^
Pooled SEM	0.43	0.35
*P*-value	0.0005	<0.0001

1 Pigment deposition is unitless; a higher number indicates greater pigment deposition. Scores are written as LS means with pooled SEM below each column. ^a-c^Means within a column lacking a common letter are different (*P* < 0.05).

2 N = 192; 32 birds/diet x litter treatment with 8 birds/replicate sampled.

## Discussion

Footpad dermatitis continues to be a widespread and serious issue within the poultry industry, affecting broilers, laying hens, turkeys, and ducks (Heerkins et al., 2016); Pagazaurtundua and Warriss, 2006; Allain et al., 2013; Klambeck et al., 2019). Footpad dermatitis negatively affects the birds, their market value, and as a growing concern, their welfare. This has prompted numerous studies focused on the prevalence and prevention of FPD (Mayne, 2005; Pagazaurtundua and Warriss, 2006; Haslam et al., 2007; Shepherd and Fairchild, 2010; Da Costa et al., 2014). While many dietary preventatives such as vitamins, minerals, amino acids, enzymes, electrolytes, and microelements supplements have been tried, no preventative solution has been found (Shepherd and Fairchild, 2010; Swiatkiewicz et al., 2017). Carotenoids are commonly supplemented to laying hens through synthetic and carotenoid-rich additives to increase yolk pigment (Moreno et al., 2016), but their use in broilers is less common. The use of supplemental carotenoids from non-GMO orange corn to prevent or reduce FPD has not been previously evaluated. Orange corn contains higher levels of carotenoids, which are believed to support skin health in humans (Juturu et al., 2016; Grether-Beck et al., 2017). In 2 different studies, 1 on rats and 1 on humans, carotenoids provided protective effects to the skin of the study subjects (Mathews-Roth and Krinsky, 1985; Lee et al., 2000). In chickens, carotenoid-enriched diets enhanced immunity through elevated antibody titers (Bédécarrats and Leeson, 2006) and decreased footpad damage during a coccidial challenge (Nogareda et al., 2016). Therefore, the hypothesis of this study was that feeding birds an orange corn–based diet, high in carotenoids, would reduce the severity of FPD in birds exposed to wet litter conditions. Using Global Animal Partnership guidelines (GAP guidelines [2017]), footpad scores of 1 and 2 correlate with mild to severe lesions, ulceration, dark papillae, or bumblefoot, while a score of 0 represents minimal or healed damage. The primary determinant of FPD severity in this study was the presence of wet litter. This is not surprising as it has been noted several times in past FPD studies, including those by Mayne (2005) and Jong et al. (2014). The results of this research also found that the orange corn diets were associated with lower severity of FPD (fewer scores of 1 and 2) especially during the latter half of this study. After 27 d of age, the incidence of moderate footpad scores decreased for birds fed orange corn but increased for birds fed yellow or white corn. Birds fed orange corn never developed scores greater than 0 on the control litter. This study found that feeding orange corn diets can reduce the severity of FPD in broiler flocks, exposed to both wet and dry litter conditions.

In addition, wet litter and/or FPD altered the composition of the cecal microbiome. Increased litter moisture likely altered the litter microbial composition (not determined), causing a change in the environmental bacteria and therefore the bacteria that was ingested and later colonized the birds' gastrointestinal tracts. Previous studies have revealed that litter plays an important role in the colonization of the chicken gastrointestinal tract (Cressman et al., 2010; Danzeisen, 2015).

This study explored whether increased carotenoid concentration (corn color) in diets would alter the microbiome composition. A slight but distinguishable shift in community membership owing to the corn source or color used to feed the birds but no change in the bacterial alpha and beta diversity. Similar to previous studies in swine (González-Prendes et al., 2019; Li et al., 2019), corn carotenoid concentration altered some bacterial genera but not community alpha diversity. Additional replicates are likely required to discern differences in the beta diversity of the microbiome owing to corn carotenoid concentration, but the magnitude of the difference is likely to be small. Given the diet caused only minimal changes to the gut microbiome (only 3 of the 1,585 ASV were statistically altered), the beneficial effects of corn carotenoid concentration is not likely mediated via changes to the gut microbiome but rather more likely through localization of carotenoids throughout the body and at the site of insult. The lack of alteration to the gut microbiome also indicates that these alternative types of corn do not induce dysbiosis or other negative results to the gut bacterial community.

Diet and litter treatment did not affect feed consumption; only phase had a significant effect on feed consumption (*P* < 0.0001; Table 3). An unexpected benefit of the orange corn diet was that it increased the birds' average BW throughout this study. This was in agreeance with a previous study of broilers fed yellow corn supplemented with Vitamin A in which the birds had increased BW from 14 d of age until conclusion of the study (Pretorius and Schönfeldt, 2013). However, a later study by Nogareda et al. (2016) found no differences in final BW of broilers fed carotenoid-enriched diets. The cause of these differences in results is unclear but may be owing to unaccounted differences in the kernel composition of the corn varieties used in the 2 studies. The increased BW in this study were likely owing to differences in nutritional content between the different corn types. The nutritional content of each of the corn sources (white, yellow, and orange) was assumed to be the same. However, after analysis of each corn source, significant differences were found. These nutritional differences in corn source should be accounted for and used to formulate future diets to meet specific strain requirements.

An unaccounted-for variable in this study was the differences in corn characteristics. The different types of corn – white, yellow, and orange – have different textural and nutritional or electrolyte traits which could influence water consumption and water content of both the feces and litter (Xu et al., 2015). The orange non-GMO corn genotype used in this study has been previously described (Ortiz et al., 2016) and has a subtropical origin containing flint-type kernels. Flinty kernels are harder and denser than dent kernels. Notably, Xu et al. (2015) showed that the dietary incorporation of 50% coarse ground corn, owing to its ability to modulate gastrointestinal function, reduced litter moisture and reduced excreta nitrogen.

Overall, the orange corn source had the highest levels of ME, CP, and crude fat (Table 2). This increased nutritional content likely contributed to the birds' increased weight gain and growth. The elevated weight gains in this study did not, however, translate into improved FCR. Feed conversion rate was affected by diet during phases 1 and 3 and was affected by litter during phase 3 only (*P* < 0.05); overall FCR was unaffected by diet and litter treatment.

In addition to improving the weight gain of the birds in this study, the orange corn diets were associated with increased pigment deposition in the liver and fat pad. Increased pigmentation and coloration can affect consumer preference and nutritional value as well as the bird's health. While carotenoids were not directly measured as part of this study, the increased pigmentation seen in the fat pads and livers likely represent increased sequestration of carotenoids as reported in previous studies (Moreno et al., 2016; Nogareda et al., 2016).

However, the mere presence of increased pigmentation and therefore increased carotenoids does not mean that deposition of all carotenoids is the same. In chickens, the liver has typically been thought of as the main storage organ for carotenoids and retinol (a breakdown product of PVA carotenoids) (Nogareda et al., 2016). But a study by Moreno et al. (2016) found that in birds, retinol and non-PVA carotenoids, such as zeaxanthin, tended to deposit in eggs when PVA carotenoids were in a surplus state. In addition, retinol and the carotenoids zeaxanthin, zeaxanthin-5,8-epoxides, and β-carotene-5,8-epoxides were elevated in the body fat of broilers fed carotenoid-enriched corn (Nogareda et al., 2016). These studies detail the importance of understanding which specific carotenoids are depositing in each organ of the chicken's body and explain why the liver and fat pad were the focus in this study. In this study, there was significant carotenoid deposition in the liver and fat pads of the birds fed orange corn as evidenced by increased colorimeter scores in these birds.

For future studies, specific carotenoids should be measured in the organs of interest, including measurements of retinol to determine bioavailability. Nogareda et al. (2016) suggested that in chickens, liver retinol could be used as a proxy for carotenoid bioavailability of a diet and that in general, carotenoids in transgenic high carotenoid corn were more bioavailable than those in commercially supplemented diets. Titcomb et al. (2018) found that carotenoids were highly bioavailable in biofortified orange corn. Their study established that in humans, *β*-cryptoxanthin and zeaxanthin were highly bioavailable in whole-grain orange corn fortified with *β*-cryptoxanthin. Providing viable biofortified products could mean improved bird and human health without the use of feed additives. In this study, the same carotenoids that resulted in increased pigment levels may have provided protection to the birds' feet or provided additional health benefits such as increased immune function, as was shown by Bédécarrats and Leeson (2006). By understanding color shifts in the fat pad and liver of the chickens in this study, by proxy, carotenoid deposition and bioavailability could be measured. However, for future studies, retinol and both PVA and non-PVA carotenoids should be directly measured in the liver, fat, and skin to determine both bioavailability of the diets and differential carotenoid deposition. Differential deposition in the meat may improve health effects for humans consuming that meat.

The highest levels of carotenoids in this study were present in the orange corn source followed by yellow and white corn (21.03, 5.36, and 0.57 μg/g, respectively. Orange corn had the highest levels of all carotenoids with the largest differences from white and yellow corn in zeaxanthin, beta-cryptoxanthin, total xanthophylls, carotenes, and PVA carotenoids. Zeaxanthin and lutein (non-PVA carotenoids) are the major carotenoids in yellow corn while β-carotene and β-cryptoxanthin (PVA carotenoids) are usually found in smaller amounts. This means that the overall vitamin A content of the diet is not increased through use of yellow corn (Pretorius and Schönfeldt, 2013). In this study, yellow corn contained only small amounts of PVA carotenoid levels. Provitamin A carotenoid levels were 0.01, 0.16, and 2.15 μg/g for white, yellow, and orange corn, respectively. So, to reap the benefits of PVA carotenoid supplementation, orange corn, which contains higher amounts of β-carotene and β-cryptoxanthin, would be required. Orange corn provides not only PVA carotenoids, but the antioxidant xanthophyll carotenoids zeaxanthin and lutein as well.

Further research should evaluate consumer opinion of increased pigmentation of the bird internally and externally. Depending on consumer preference, the altered appearance, in the form of yellower skin, fat, and organs, may affect the marketability of birds fed orange corn diets. Williams reported in 1992 that historically Americans have preferred chickens with higher pigment color (Williams, 1992). Nogareda et al. (2016) noted that golden skin color is generally preferred but that these preferences vary by geographical region. Therefore, chicken pigmentation preferences specific to a region would provide beneficial information for marketing. In addition, increased pigmentation and increased carotenoids may improve health effects in the humans consuming them, but this requires further evaluation.
